# First experimental demonstration of real-time neutron capture discrimination in helium and carbon ion therapy

**DOI:** 10.1038/s41598-024-52162-9

**Published:** 2024-01-31

**Authors:** Marissa Kielly, Anita Caracciolo, Andrew Chacon, James Vohradsky, Davide Di Vita, Akram Hamato, Hideaki Tashima, Daniel R. Franklin, Taiga Yamaya, Anatoly Rosenfeld, Marco Carminati, Carlo Fiorini, Susanna Guatelli, Mitra Safavi-Naeini

**Affiliations:** 1 Australian Nuclear Science and Technology Organisation (ANSTO), Lucas Heights, Australia; 2 Centre for Medical Radiation Physics, University of Wollongong, Wollongong, Australia; 3 Dipartimento di Elettronica, Informazione e Bioingegneria, Politecnico di Milano, Milan, Italy; 4 Istituto Nazionale di Fisica Nucleare (INFN), Sezione di Milano, Milan, Italy; 5 Imaging Physics Group, Department of Advanced Nuclear Medicine Sciences, National Institutes for Quantum Science and Technology (QST), Inage-ku, Chiba, Japan; 6 School of Electrical and Data Engineering, University of Technology Sydney, Sydney, Australia

**Keywords:** Biological physics, Particle physics, Biomedical engineering, Translational research

## Abstract

This work provides the first experimental proof of an increased neutron capture photon signal following the introduction of boron to a PMMA phantom during helium and carbon ion therapies in Neutron Capture Enhanced Particle Therapy (NCEPT). NCEPT leverages ^10^B neutron capture, leading to the emission of detectable 478 keV photons. Experiments were performed at the Heavy Ion Medical Accelerator in Chiba, Japan, with two Poly(methyl methacrylate) (PMMA) targets, one bearing a boron insert. The BeNEdiCTE gamma-ray detector measured an increase in the 478 keV signal of 45 ± 7% and 26 ± 2% for carbon and helium ion irradiation, respectively. Our Geant4 Monte Carlo simulation model, developed to investigate photon origins, found less than 30% of detected photons originated from the insert, while boron in the detector’s circuit boards contributed over 65%. Further, the model investigated detector sensitivity, establishing its capability to record a 10% increase in 478 keV photon detection at a target ^10^B concentration of 500 ppm using spectral windowing alone, and 25% when combined with temporal windowing. The linear response extended to concentrations up to 20,000 ppm. The increase in the signal in all evaluated cases confirm the potential of the proposed detector design for neutron capture quantification in NCEPT.

## Introduction

Neutron Capture Enhanced Particle Therapy (NCEPT) is a new form of radiotherapy that enhances the dose delivered during particle therapy by capturing internally-generated thermal neutrons in cancer cells. This is achieved by injecting tumour-specific neutron capture agents (NCAs) based on high thermal neutron cross-section isotopes such as ^10^B or ^157^Gd, which preferentially accumulate in cancer cells. Neutrons generated via nuclear fragmentation processes which occur during particle therapy are thermalised in the body and captured by the agent, releasing high linear energy transfer (LET) particles which deliver an additional dose to the target. The neutron fluence in the target varies with its size, depth, and composition, while the resulting dose is dependent on the spatial distribution of the NCA^1^. Although therapeutic neutron capture is known to be feasible with ^10^B-based agents and expected to be feasible using agents based on ^157^Gd^2,3^, boron-based agents such as L-boronophenylalanine (BPA) and sodium mercaptoundecahydro-closo-dodecaborate (BSH) are much more mature than gadolinium-based agents and several are already approved for clinical use^4,5^.

While several approaches have been proposed for dose quantification for quality assurance in particle therapy, for example using positron emission tomography (PET), single photon emission computed tomography (SPECT) or prompt gamma imaging^6–9^, none of the existing methods are directly applicable to NCEPT as they do not quantify the dose component resulting from neutron capture. Similarly, dose quantification methods from boron neutron capture therapy (BNCT) are not directly applicable either, since NCEPT operates in a far more complex radiation field, including ions, neutrons and gamma photons across a wide range of energies. To address this, it is critical that an in-vivo system is developed for monitoring both ion and neutron capture dose components. One potential approach is through the detection of characteristic photons emitted during neutron capture; for ^10^B these photons are emitted with a single spectral peak at 478 keV.

The gamma spectrum emitted from the target region during NCEPT is very similar to that which is produced during conventional particle therapy, featuring a background continuum and prompt gamma spectral peaks at characteristic energies—including the positron annihilation peak at 511 keV and a 2.2 MeV hydrogen neutron capture peak^10–13^. Neutron capture peaks resulting from ^10^B (or ^157^Gd) neutron capture are also present in the photon spectrum in NCEPT. The proximity of the ^10^B neutron capture peak at 478 keV to the 511 keV positron annihilation peak necessitates the use of a detector with high energy resolution (better than 3% at 662 keV) to reliably discriminate between these two peaks. Further complicating the task of neutron capture quantification is the complexity of the radiation field; the multitude of scattered ions and fast neutrons from the beam, fragments resulting from nuclear interactions in the target, and neutron activation of both the detector and shielding all contribute to the background from which neutron capture events must be unambiguously discriminated.

Prompt gamma imaging is a technique used to evaluate the distribution of high-energy photon emission resulting from the de-excitation of nuclei from short-lived excited states, such as those resulting from neutron capture. It has been studied extensively for potential use in particle therapy range verification, with experimental and simulation studies conducted by multiple groups^10,14–16^. In particular, the distribution of 478 keV photons produced during boron neutron capture therapy (BNCT) has been investigated using prompt gamma—single photon emission computed tomography (PG-SPECT) systems for dose quantification and imaging^17^. While this general approach is well-suited to BNCT, its applicability to NCEPT is greatly compromised by the complexity of the radiation field; dosimetry is also more complex since there are two distinct means of dose deposition in the target (ion dose and neutron capture dose). Furthermore, due to the low concentrations of boron that will be delivered to the patient during treatment, the successful implementation of prompt gamma imaging in NCEPT requires the ability to detect relatively small changes in the high background signal.

In our previous Monte Carlo simulation study, we have demonstrated that discrimination between neutron capture photons and other photon or particle detection events is theoretically feasible based on a combination of spectral and temporal windowing, with the latter being performed relative to the ion beam pulse structure^18^. In this study, we experimentally quantify the neutron capture photons emitted during carbon and helium ion irradiation of a PMMA target with and without a boron-loaded insert, and compare the results to the output of an equivalent Monte Carlo simulation. We are only considering boron in this work as the prototype detector is optimised for detection of 478 keV photons. The first-generation prototype detector does not have temporal windowing capabilities, however it is expected that the addition of this feature would further improve the selectivity of the system; therefore, the performance of a realistic model of the detector is additionally evaluated with and without temporal windowing in simulation. Finally, we examine the performance of our proposed system in response to changes in the concentration of ^10^B in simulation.

This paper consists of the following sections: the experimental configuration and methods are described in “Materials and Methods” Section; results are presented in “Results” Section and discussed in "Discussion" Section; finally,  “Conclusions” Section then gives a summary of our findings and conclusions, and discusses the future work arising from this project.

## Materials and methods

All experimental measurements were performed using the Heavy Ion Medical Accelerator in Chiba (HIMAC) biological beamlines at the National Institute for Quantum Science and Technology (QST) in Japan. Photon measurements were conducted using the BeNEdiCTE (Boron NEutron CapTurE) gamma-ray detector developed at Politecnico di Milano^19^. This detector is designed to be able to discriminate between the 478 keV photons resulting from ^10^B neutron capture and the adjacent 511 keV positron annihilation photopeak, which is essential for being able to quantify the enhanced neutron capture dose in NCEPT. The simulations in this study, conducted using the well-known Geant4 Monte Carlo toolkit^20,21^, model the detector with a simplified representation of the experimental configuration.

### Experimental configuration

**Figure 1:**
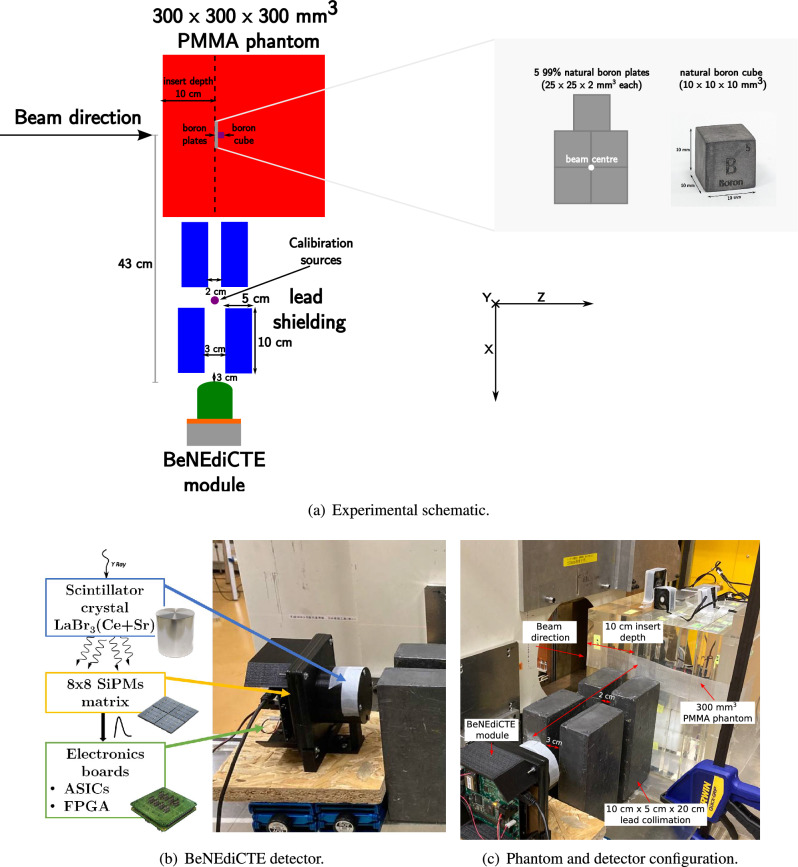
Experimental configuration.

The experimental configuration, consisting of a phantom, detector and lead collimator, is shown in Fig. 1a. The full details of the BeNEdiCTE system can be found in the paper by Caracciolo et al.^19^; however, its design, composition and essential properties are briefly summarised here. The module is based on a cylindrical LaBr_3_ scintillator crystal, 2 inches (50.8 mm) in diameter and 2 inches thick, co-doped with cerium (Ce) and strontium (Sr) and coupled to an $8 \times 8$ array of silicon photomultipliers (SiPMs). The detector exhibits an energy resolution of 2.7% at 662 keV and the crystal provides a detection efficiency of 90% at 478 keV^19^. SiPM readout is performed with four custom application-specific integrated circuits (ASICs), and data acquisition is controlled by a field-programmable gate array (FPGA). This dedicated electronics is fabricated on printed circuit boards (PCBs) placed just beneath the crystal (see Fig. 1b). The PCBs are made of FR-4 material, which is a composite of epoxy resin and “E”-grade glass fibre. Unfortunately, approximately 0.2-0.3% of the mass of this material is ^10^B^22^. Due to the presence of ^10^B, there is a background signal of 478 keV photons which has been previously reported for this detector^23^.

The experiments in this study were conducted with 60 mm carbon and helium spread out Bragg peak (SOBP) ion beams produced at HIMAC’s biological beamline, with a depth range of 85–145 mm in PMMA and a 10 cm $\times$10 cm field size. For each irradiation, 0.33 Gy was delivered to the SOBP.

Two 300 mm cubic PMMA phantoms were constructed. For the first phantom, five 25 mm $\times$ 25 mm $\times$2 mm natural boron plates were arranged in a flat plane (see Fig. 1b) at a depth of 100 mm, normal to the beam and centred on the beam axis. A 1 cm cube of natural boron was additionally placed along the central axis of the beam directly distal to the plates. The positions of the boron plates and cube were selected to coincide with the approximate position of maximum expected neutron flux^1,24^, and aligned to the aperture of the collimator, such that the number of neutron capture photons arriving at the detector would be maximised (Fig. 1a).

The detector was oriented perpendicular to the beam and aligned with the boron inserts at a lateral offset of 43 cm from the beam centre (see Fig. 1c) This distance was chosen so as to place the detector behind the shielding structure housing the brass collimator which trims the beam field size.

Four lead blocks were added for collimation of photons exiting the phantom, each measuring 10 cm $\times$ 5 cm$\times$ 20 $\times$ cm. Two of these blocks were situated adjacent to the phantom, while the remaining two blocks were positioned 3 cm from the detector, as shown in Fig. 1a,c. The collimation aperture for the pair adjacent to the phantom was 2 cm, while the aperture for the second pair was 3 cm to expose the entire detector face.

Data was acquired only during the irradiation period, which totalled 10.5 min. for each measurement. For the calibration of the detected spectra, ^137^Cs (662 keV) and ^133^Ba (303 keV and 356 keV) standard sources were placed on the table at the midpoint of all four lead collimators; this position was chosen so as to enable consistent placement of the calibration sources between experiments, and to provide simultaneous visibility of both the target region and the calibration sources to the detector without causing an excessively high count rate from the calibration source.

### Monte Carlo simulations

A model of the beam, target, collimator and detector described in the previous section was constructed in Geant4 version 11.0^20^. Geant4 has been extensively validated for medical physics applications, especially with regards to gamma photon transport and the yield of positron-emitting secondary fragments^21,25^. The material properties for each component are as defined in the National Institute of Standards and Technology (NIST) database in Geant4^26^. Electromagnetic interactions were modelled using G4EmStandardPhysics_option4; for neutron interactions the High Precision model is used, while the Binary Ion Cascade (BIC) model was selected for hadronic inelastic nuclear interactions. The full table of physics models implemented in Geant4 is given in the Supplementary Materials as Table S1.

To cross-validate the neutron spectral and spatial distributions produced via Geant4 in this work, a series of additional simulations were conducted using MCNP 6.2, a completely independent and well-regarded Monte Carlo platform for the simulation of particle transport in matter (especially neutrons)^27,28^. Spectra of neutrons leaving the phantom and arriving at the detector face were compared in simulations performed using both MCNP 6.2 and Geant4 11.0. Details of these simulations, including physics models, geometry (Fig. S1) and reported results (Figs. S2 and S3), are presented in Supplementary Materials.

Ion beams were simulated with energies ranging from 225 to 294 MeV/u for carbon and 113 to 156 MeV/u for helium, producing a 60 mm SOBP across the same depth range as used in the experiments. The procedure used to construct the spectra for these beams is as described by Safavi-Naeini et al.^1^. The beam dimensions were set to 10 cm $\times$ 10 cm, and a total of $2\times 10^{10}$ and $4\times 10^{10}$ particle histories were simulated for the carbon and helium ion beams, respectively.

In addition to the natural boron which was used in the experiment, ^10^B-loaded inserts were also simulated with a range of concentrations to investigate how this affects the amplitude of the neutron capture photon signal. For these simulations, a total of $4\times 10^{9}$ particles were simulated for both the carbon beam and helium ion beams. The insert shape and position remained the same, with the compositions as follows:

- Pure (100%) ^10^B
- PMMA with 100000 ppm ^10^B
- PMMA with 10000 ppm ^10^B
- PMMA with 5000 ppm ^10^B
- PMMA with 1000 ppm ^10^B
- PMMA with 500 ppm ^10^B

As our previous work has highlighted the advantage of implementing temporal windows to discriminate the neutron capture photons, this was also considered here through the inclusion of a timing window on all photons depositing energy in the detector. We have previously shown that neutron capture photons begin to arrive at the detector from 22 ns, with thermal neutrons from the phantom arriving after 10^4^ ns^18^. As such, this window was also considered here.

The walls and other equipment were not modelled in this simulation, as it is assumed that the number of detected events which may occur due to neutron capture and scattering in the room will be consistent between the boron and no-boron cases. The subtraction process for background removal (discussed in the following section) is assumed to remove this contribution and hence it should not affect the observed difference. Similarly, the fast neutron component originating from the beamline and background radiation in the room were not explicitly modelled.

All energy deposition events inside the LaBr_3_:Ce/Sr detector were recorded, with the originating volume, arrival time and energy of the particle being scored. The optical scintillation process is not modelled; the output signal is assumed to be directly proportional to the energy deposited in the detector. Analysis was performed only for those energy deposition events which occurred inside the detector during the irradiation period. Simulation time was measured relative to the time of generation of the primary particles at the surface of the phantom, with a total irradiation time of 10.5 min.

### Comparison of simulation and experimental spectra

The gamma-ray spectra obtained experimentally and in simulation for the boron and no-boron configurations was analysed in MATLAB. For the simulations, the energy resolution of the detector was modelled by convolving the energy spectrum with Gaussian functions whose spread was based on the measured energy resolution of the experimental prototype (with these measurements performed as part of the pre-beam calibration procedure): the full width at half maximum (FHWM) for each of the calibration peaks (303 keV and 356 keV for ^133^Ba and 662 keV for ^137^Cs) was found and a linear interpolation/extrapolation was performed to estimate the energy resolution of the detector for all energies.

#### Removal of background continuum

The background continuum of scattered photons needs to be accounted for in the analysis of both the experimental and simulation spectra. A simple analytic model consisting of a linear term plus two Gaussians - one centred on 511 keV (the positron annihilation peak, which we have previously shown to be almost entirely due to positrons created by the decay of positron-emitting nuclear fragments^25^) and the other on 478 keV (the boron neutron capture peak)—is fitted to the observed spectra via the trust-region-reflective algorithm as implemented in MATLAB’s lsqnonlin function^29^. This allows estimates of the contribution of each peak to be separated from the observed background continuum of the spectrum. The model is described by (1):

1 $$\begin{aligned} y = b + mx + \frac{A_1}{\sigma _1 \sqrt{2\pi }}exp\left( {-\frac{(x - E_{capt})^2}{2\sigma _1^2}}\right) + \frac{A_2}{\sigma _2 \sqrt{2\pi }}exp\left( {-\frac{(x - E_{ann})^2}{2\sigma _2^2}}\right) \end{aligned}$$

where *b* and *m* are the parameters of the assumed linear background continuum, $A_{1,2}$ are the amplitudes of the two Gaussian peaks, $E_{capt}$ and $E_{ann}$ are the energies of the capture and positron annihilation peaks, respectively (478 keV and 511 keV), and $\sigma _{1,2}$ are the standard deviation parameters of the two Gaussians. The assumption of a linear spectral continuum is only valid over a narrow range of energies and cannot be extrapolated outside of this range, however it is adequate for the purpose of pedestal removal at the energies of interest (478 keV and 511 keV).

The positron annihilation peak was included in this model due to its proximity to the neutron capture photopeak and for correct scaling of the final spectra. In previous work we have shown that the vast majority of 511 keV photons are a result of positrons created by the decay of positron-emitting fragments^25^.

**Figure 2:**
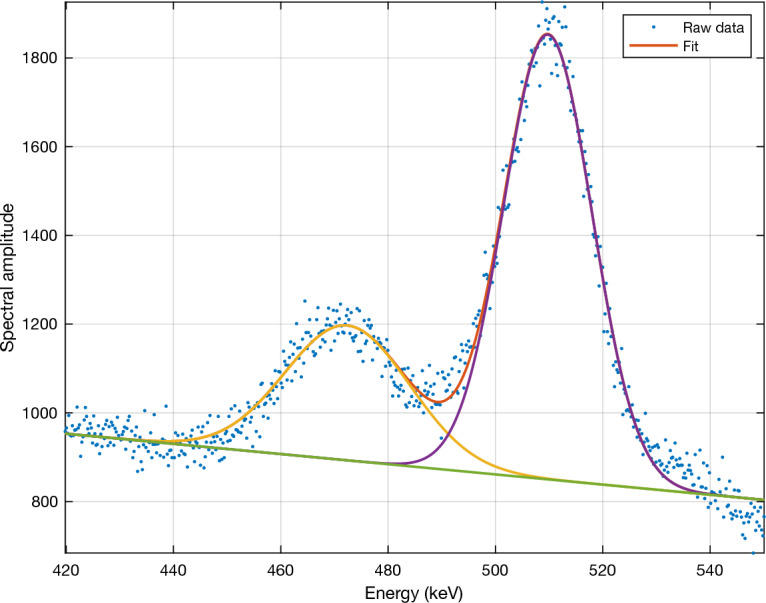
The sum of a linear and two-Gaussian fit for a spectrum obtained from experimental measurements performed with the prototype detector.

This approach is a simplified version of the multi-energy-window interpolation method^30^ and is illustrated in Fig. 2. The fit is applied to an energy range wide enough that the contribution from the 478 keV and 511 keV peaks is negligible at the upper and lower end of the range. The lower energy was chosen to coincide with the minimum before the 478 keV neutron capture peak (although any energy in the range 400–440 keV could be used). The upper energy was chosen to be at 550 keV, which is above the upper extent of the 511 keV positron annihilation peak.

#### Spectral analysis and quantification of changes in photon count with boron present in the target

Energy spectra were accumulated following simulated and experimental irradiation with both helium and carbon ion beams, for targets configured with and without the boron inserts. Acquired spectra were conditioned (i.e., the pedestal was removed) and normalised to the height of the fitted 511 keV Gaussian peak for each data set. This peak was used for normalisation since its height is independent of the presence or absence of ^10^B in the target. Average experimental heights and areas of each peak, as well as the respective standard errors of mean were calculated over three repetitions of each experiment.

For the simulation results, 20 runs with different random seeds were generated, and the inter-run average and standard deviation of the height or area under the curves following background subtraction were calculated. All error bars in this paper are shown for confidence intervals of ± 2 standard errors of mean at 10 keV energy increments and quantitative values are also presented with these uncertainties.

The photon signal due to neutron capture was quantified through the ratios of both the height and area of the 478 keV peak to those of the 511 keV peak. These ratios, for both the boron and no-boron cases, are then compared to quantify the increase which occurs due to neutron capture inside the insert. The areas under the curves were calculated from the variables obtained during the scatter correction and Gaussian fit performed as discussed in the preceding section, both for the experimental and simulated results (with the Gaussian convolution applied).

### Volumes of origin of detected photons

In each simulation (helium/carbon, with boron insert absent/present), the volume of origin of each particle which deposited energy within the detector was recorded. This enables the total spectrum of energy deposited in the detector to be decomposed into the components originating from different elements of the simulation—in particular, the contributions of 478 keV photons from neutron capture events in the insert region inside the phantom and the PCB in the detector. These spectra have also been convolved with a Gaussian filter to mimic the energy resolution of the detector as previously discussed, with results normalised to the 511 keV peak height for all counts. The contributions from the PCB only and the PCB+insert to the total photon spectrum are plotted separately to better illustrate these components.

Additionally, the heights of the 478 keV peak in the raw simulated spectra (i.e. prior to the Gaussian convolution) are compared to determine the percentage of the total signal which comes from each volume. For each set, the total number of 478 keV photons from the insert and PCB is given as a percentage of the total.

### Neutron capture signal with respect to ^10^B concentration

For each concentration of ^10^B considered in the simulation, the energy deposition events inside the detector were recorded. As with the simulated results obtained for the natural boron insert, the energy resolution of the detector was modelled by the energy-dependent convolution method. The spectrum obtained for the irradiation period was then fitted to a two-Gaussian model as discussed previously and the neutron capture photon signal is quantified through the height and area of the neutron capture peak relative to the 511 keV positron annihilation photopeak. Error bars indicate a range of $\pm \sqrt{N}$, where *N* is the number of counts.

To demonstrate the improvement in specificity which can be achieved by adding temporal windowing, the simulation output is filtered to the time interval between 22 ns and $10^4$ ns post-irradiation (which was found to be optimal in our previous publication^18^). As most of the positron annihilation photons arrive at the detector after 10^4^ ns, the area under the neutron capture peak was considered independently for the results with this timing window through a single Gaussian fit.

Both sets of data are fitted to a simple asymptotic regression model to account for the initial linear response and plateau at higher concentrations. This model is fitted to the following equation:

2 $$\begin{aligned} y = a - (a-b)exp(-cx) \end{aligned}$$

where *x* is the concentration as a percentage of ^10^B and the constants *a*, *b* and *c* are obtained through the fitting process.

Finally, the minimum ^10^B concentration at which neutron capture events can be reliably detected is estimated for both the spectral-only and spectral/temporal windowing cases.

## Results

Simulation results cross-validating the Geant4 neutron physics models against those of MCNP 6.2 are presented in Supplementary Materials. Figures S2 and S3 show the neutron spectra obtained from simulated irradiation of the phantom by helium and carbon ions in MCNP 6.2 and Geant4, both exiting the phantom surface and arriving at the detector surface. The ratios of the total number thermal neutrons leaving the phantom and arriving at the detector surface predicted by MCNP to the totals predicted by Geant4 are 0.89 (leaving) and 0.72 (arriving) for helium and 1.12 (leaving) and 0.97 (arriving) for carbon (summarised in Table S1).

### Quantification of photon count increase due to boron neutron capture

**Figure 3:**
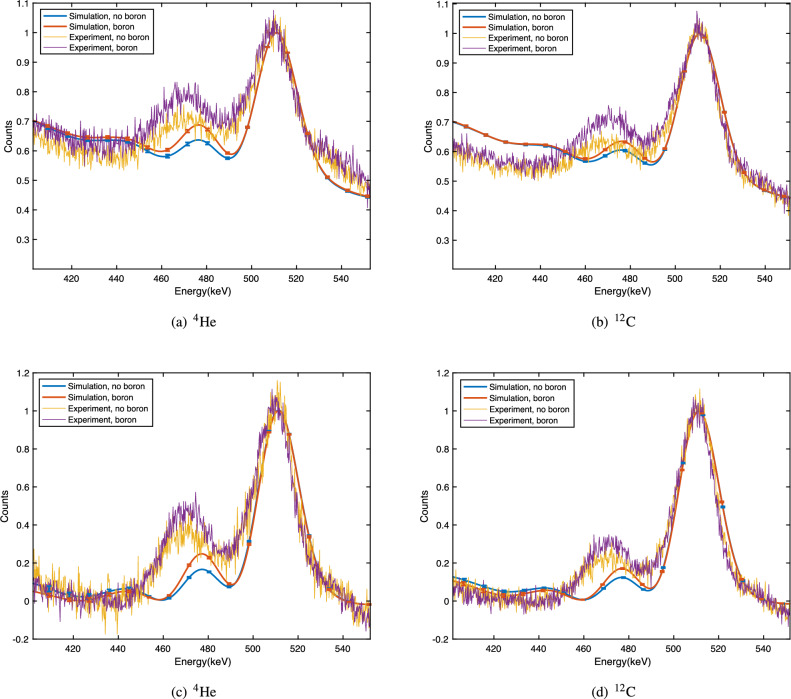
Spectra of energy deposited inside the detector for each beam type, showing simulation results with and without boron overlaid on top of the experimental measurements; the energy range (*x*-axis) is restricted to 400–550 keV. One representative pair of experimental results has been plotted for each case and all values are scaled to the height of the 511 keV peak, with the pedestal removed prior to normalisation in (**c**) and (**d**).

The energy spectra obtained in the experimental measurements are plotted together with the corresponding simulation results in Fig. 3 for both helium and carbon ion beams. Figure 3a,b present the spectra with normalisation prior to pedestal removal, while Fig. 3c,d were normalised following this. This process was employed for quantitative analysis in the following section. An increase in the neutron capture photon signal with the addition of the boron insert can be observed for all cases.

Table 1 presents the experimental and simulation-based heights and areas of the neutron capture peak relative to the 511 keV annihilation peak with the boron insert present or absent during irradiation with the ^4^He ion beam, while corresponding values for the ^12^C beam are shown in Table 2. The percentage increase for each case is also shown.

**Table 1:** Intensity of the 478 keV peak with and without a boron insert with the ^4^He beam. Each total is normalised to the corresponding value for the 511 keV positron annihilation peak.

	Height		Area	
	Experiment	Simulation	Experiment	Simulation
No boron	0.33 ± 0.01	0.174 ± 0.003	0.50 ± 0.02	0.126 ± 0.004
Boron	0.48 ± 0.02	0.258 ± 0.005	0.63 ± 0.03	0.198 ± 0.005
Increase (%)	39 ± 2	48 ± 1	26 ± 2	57 ± 1

**Table 2:** Intensity of the 478 keV peak with and without a boron insert with the ^12^C beam. Each total is normalised to the corresponding value for the 511 keV positron annihilation peak.

	Height		Area	
	Experiment	Simulation	Experiment	Simulation
No boron	0.211 ± 0.006	0.131 ± 0.003	0.35 ± 0.01	0.092 ± 0.002
Boron	0.33 ± 0.03	0.179 ± 0.003	0.51 ± 0.08	0.134 ± 0.003
Increase (%)	58 ± 5	36 ± 1	45 ± 7	45 ± 1

From the total area under the 478 keV peak when the boron insert was present, the number of energy deposition events recorded with the prototype detector within the neutron capture peak energy range increased by 26 ± 2% for the helium ion beam and 45 ± 7% for the carbon beam relative to the no-boron case. In simulation, the corresponding number of events increased by 57 ± 1% and 45 ± 1% for the helium and carbon ion beams, respectively.

The height of the neutron capture photon peak increased by 39 ± 2% and 48 ± 1% for the experimental and simulated helium ion beams, respectively, while for carbon the heights increased by 58 ± 5% experimentally and 36 ± 1% via simulation.

### Volumes of origin of detected photons

**Figure 4:**
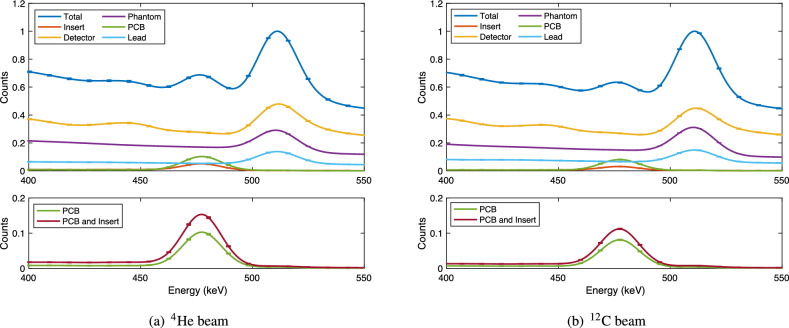
Total energy deposition in the detector decomposed according to the volume in which the photon originated. The upper plots show the result for all simulated volumes, while the lower plots show only the contributions from the detector PCB and insert (lower plot); the energy range (*x*-axis) is restricted to 400–550 keV and all values are scaled to the height of the 511 keV peak.

Figure 4 shows the total photon spectra as observed in the detector and the decomposition of this spectrum into the contributions originating from each volume, for the simulations with the boron insert present. In each case, the boron plates and cube are considered to be a single volume, named “insert” in the figure.

These figures show that the majority of the energy deposited inside the detector in the range 400–550 keV comes from photons generated in the detector itself. This is then followed by photons emitted from the phantom and the lead collimators. For both ion beams, the 478 keV peak for the insert is smaller than the contribution from the lead collimator; the peak of the 478 keV contribution from the PCB slightly exceeds that from the collimator.

For the helium ion beam, 29 ± 2% of the total neutron capture photons at the 478 keV peak (without convolution) came from the insert, while 66 ± 2% originates in the PCB. With the carbon ion beam, the percentage of neutron capture photons from the insert is 24 ± 1% and for the PCB it was 69 ± 1%.

### Neutron capture signal with respect to ^10^B concentration

**Figure 5:**
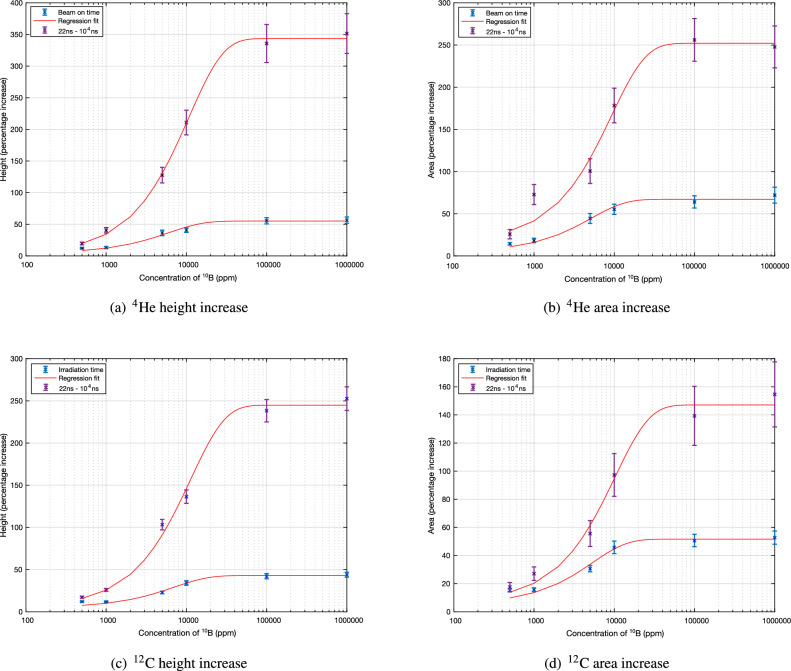
Increase in the neutron capture photon signal (height and area) with concentration for each ion beam. The percentage increase over the 10.5 min. irradiation time is in blue, while the results with the temporal window are given in purple.

The change in the neutron capture photon signal as the concentration of ^10^B is increased, quantified through the relative change in height and area of the neutron capture peak (normalised to the positron annihilation peak), are given in Table 3 for the helium ion beam and Table 4 for the carbon ion beam. This is also visualised in Fig. 5, along with the asymptotic regression fit. The increase in the capture signal relative to the no-boron case when a 22 ns to 10^4^ ns temporal window is applied to the simulation output is also shown.

**Table 3:** Intensity of the 478 keV peak with increasing concentration for the simulated results with the ^4^He beam, over both the full irradition time of 10.5 min. and a shorter temporal window of 22 ns-10^4^ ns. The percentage increase is relative to the no-boron case.

	Height increase (%)		Area increase (%)	
	Full irradiation time	22 ns-10^4^ ns only	Full rradiation time	22 ns-10^4^ ns only
500 ppm	12.2 ± 1.0	19.6 ± 2.2	14.4 ± 1.4	25.8 ± 5.6
1000 ppm	13.2 ± 1.6	40.6 ± 4.3	18.3 ± 2.6	72.8 ± 12.0
5000 ppm	36.4 ± 4.4	128 ± 12	44.5 ± 5.9	101 ± 15
10000 ppm	40.7 ± 3.5	211 ± 20	55.3 ± 6.1	178 ± 20
100000 ppm	55.6 ± 4.8	336 ± 39	64.1 ± 7.2	256 ± 25
1000000 ppm (100%)	56.5 ± 5.0	352 ± 31	72.0 ± 9.4	248 ± 25

**Table 4:** Intensity of the 478 keV peak with increasing concentration for the simulated results with the ^12^C beam, over both the full irradition time of 10.5 min. and a shorter temporal window of 22–10^4^ ns. The percentage increase is relative to the no-boron case.

	Height increase (%)		Area increase (%)	
	Full irradiation time	22 ns-10^4^ ns only	Full irradiation time	22 ns-10^4^ ns only
500 ppm	12.1 ± 0.8	17.3 ± 1.2	15.3 ± 1.2	17.6 ± 3.2
1000 ppm	11.8 ± 0.8	25.9 ± 1.7	15.7 ± 1.3	27.1 ± 4.8
5000 ppm	22.8 ± 1.5	103.3 ± 6.1	30.7 ± 2.3	55.6 ± 9.2
10000 ppm	34.1 ± 2.7	136.4 ± 7.9	45.8 ± 4.4	97 ± 15
100000 ppm	42.2 ± 3.1	238 ± 13	50.6 ± 4.4	139 ± 21
1000000 ppm (100%)	43.9 ± 3.0	253 ± 14	52.7 ± 4.7	155 ± 23

An increase in both the height and area of the neutron capture photon peak with increasing concentration of ^10^B is measurable for both ion beams; this increase is more pronounced with the helium beam, as was the case in the previous boron/no-boron study.

The percentage increase for a pure boron insert compared to the no-boron case was found to be 72.0 ± 9.4 for the helium ion beam, and 52.7 ± 4.7 for the carbon ion beam. This increases greatly with the addition of the timing window to 248 ± 25 and 155 ±  3 for helium and carbon ion beams, respectively.

## Discussion

In this study, both experimental and simulation data demonstrate a measurable increase in the neutron capture photon signal following the introduction of boron into the target volume. The magnitude of the neutron capture peak observed in the simulation is less pronounced than that observed experimentally (Fig. 3). This discrepancy suggests that Geant4 may underestimate the number of neutrons generated by the particular combination of ion beams and target material used in this study. Previous experimental investigations into thermal neutron yield in carbon and helium ion therapy corroborate this interpretation^24^; here, we additionally performed a quantitative comparison between MCNP and Geant4 regarding neutron production and transport. The differences between both the spectra of neutrons exiting the phantom and arriving at the detector are primarily in the fast neutron energy range, at energies above 1 MeV (with a maximum pointwise difference of 28% at 10 MeV for ^4^He); MCNP and Geant4 produce very similar results in the thermal/epithermal energy range (the left-hand peak in Supplementary Materials Figs. S2 and S3). Neutrons with energies $>> 1$ eV will not impact the boron neutron capture reaction in the target, although they may still deposit energy in the detector or cause activation of materials.

A confounding factor that may also partially explain the discrepancies observed between simulation and experimental spectra is the fact that several potential sources of scattering (such as the primary beam scatterer used for dose-rate adjustment, and other objects which are present in the room during the experimental measurements) are not included in the simulation model. This results in an underestimation of the background of thermal neutrons arriving at the PCB in the simulation relative to the experiment, with the effect of causing the component of the 478 keV photon peak due to neutron capture in the detector PCB to be underestimated in the simulation (Fig. 4).

Additionally, the noticeable difference in the width of each peak for the simulated spectra in comparison to the corresponding experimental results may be due to changes in the energy resolution of the detector during irradiation; the simulated spectra were convolved with the energy resolution of the detector as measured at the beginning of the night, and detector resolution is known to be dependent on temperature. While the SiPMs of the detector feature gain adjustment with variations in temperature, some small changes in resolution can still occur. In the future, energy resolution will be monitored throughout the experiments and any variation will be taken into account for analysis and comparison to simulation results.

Our previous publication evaluated the expected temporal characteristics of neutron capture photons relative to the timing structure of the ion beam and discusses the possibility of using a combination of temporal and spectral windowing for discrimination of the neutron capture photons from other prompt gamma and delayed emissions, such as positron annihilation photons^18^. As temporal discrimination is yet to be incorporated into the prototype detector system experimentally, the observation of a measurable increase in 478 keV neutron capture photons with both helium and carbon ion beams using spectral techniques alone is a promising indication that the complete system will be effective.

Tables 1 and 2 indicate the magnitude of the increase in the boron signal following the addition of boron. The percentage increase in height for both beams show close agreement between the simulation and experimental results. The increase in area with boron for the carbon ion beam also matches the experimental increase. While the increase in area with the helium beam in simulation is larger, the shape of the normalised spectra (Fig. 3) is quite similar in the simulation and experiment for both helium and carbon beams. Differences between the simulated and experimental background photon and neutron fluences (due to scattering from objects in the room, in the experimental case) may explain variations observed in this study.

The smaller relative simulation neutron capture peak heights (such as 0.258 ± 0.005 and 0.179 ± 0.003 for the simulation versus 0.48 ± 0.02 and 0.34 ± 0.03 for the experimental spectral peaks with boron for helium and carbon ions, respectively) are likely to be attributable to the known Geant4 underestimation of thermal neutron production^24^.

In both the simulation and experiment, the intensity of the spectral peaks observed for the helium ion beam prior to normalisation was greater than for the corresponding carbon ion beam, since to deliver a given dose, the helium ion beam requires a greater number of ions compared to carbon - hence there are more interactions overall.

The ratio of the neutron capture peak height to that of the positron annihilation photons is higher for the helium beam, at 0.48 ± 0.02 compared to 0.33 ± 0.03 for carbon in the experimental results with the boron insert. For the simulated spectra, this is 0.258 ± 0.005 and 0.179 ± 0.003 for the helium and carbon ion beams, respectively. This can be attributed to the number of positrons in each case; no positron emitting fragments will be created as a result of helium ion fragmentation, therefore the 511 keV annihilation peak is lower in magnitude for this beam. As such, it is important to note that inter-species comparison of the boron and no-boron ratios in Tables 1 and 2 cannot be directly made due to this difference.

The ratio of peak area to height (which is proportional to the $\sigma$ parameter of a Gaussian function) is consistently larger for the experimental results (1.3–1.7) compared to the simulation (0.70–077), despite the simulated spectra being convolved with a unit Gaussian with its $\sigma$ equal to the measured energy resolution of the physical detector. This is due to the use of the ^137^Cs calibration source, which was left in situ during the experiment to monitor changes in detector gain and energy resolution over the course of the experiment. The Compton edge of a 662 keV gamma source is located at precisely 478 keV. The presence of this source in the experiment has the effect of adding additional bulk to the left-hand side of the 478 keV neutron capture peak, with the consequence that the peak is broadened and its peak energy shifted slightly leftward (as can be seen in Fig. 3). This effect could not be precisely replicated in the simulation since the exact position of the calibration source was not recorded. It is important to note that this observation does not impact the fundamental conclusion of the paper—that this detector is able to detect the 478 keV photons resulting from neutron capture and quantify the increase in the peak amplitude due to the insertion of the boron bolus in the target phantom. In future experiments the ^137^Cs calibration source will only be used immediately before and after beam-on measurements.

The simulation-based investigation of the volume of origin for all detected photons (Fig. 4) demonstrates that a relatively small fraction of the neutron capture photons which reach the detector originate from the insert, with the dominant contribution being due to neutron capture in boron within the PCB on which the detector electronics is mounted, due to its proximity to the scintillator crystal. The fraction is larger for the case of the the helium ion beam than for the carbon ion beam. This observation demonstrates the importance of shielding around the detector and suggestions the use of alternative PCB materials fabricated from boron-free materials. Separating the detector crystal from the electronics to increase the distance between the PCB and the beam may also be an option, however this would require longer wires and hence could contribute to a loss of signal.

In total, 29 ± 2% and 24 ± 1% of the detected photons in the 478 keV photon peak originated in the insert for helium and carbon ion irradiation, respectively. The greatest net contributions to the energy spectrum of detected photons in the vicinity of the neutron capture peak are the component originating in the detector volume itself, followed by Compton-scattered photons from the lead collimator and then photons originating inside the phantom but not from the insert, i.e., not due to boron neutron capture. In this experiment, since the detector, collimator and phantom components remain in place where the boron insert is either present or absent, the background can be subtracted to obtain the contribution arising from the boron insert alone . In a practical SPECT-like neutron capture imaging system based on a detector similar to BeNEdiCTE, the background may vary significantly from detector to detector - however, using the scatter-correction method previously described, the contribution from boron neutron capture should remain separable.

A progressive increase in the neutron capture photon signal is measurable with increasing concentration of ^10^B up to around 20,000 ppm, where the magnitude of this signal reaches a plateau. This plateau can be attributed to the number of neutrons thermalised inside the phantom; at a concentration of approximately 20,000 ppm, there are no longer any thermal neutrons remaining for neutron capture inside the target. At the lower end of the concentration range, an increase in signal of 10% can be observed with spectral windowing only at concentrations of 500 ppm, improving to 25% when temporal windowing is also employed (note: the nonlinearity which is observed at low concentrations is likely due to the relatively low statistics obtained in this range). Extrapolating to lower concentrations suggests that the combined spectral and temporal windowing approach should be able to obtain a signal increase of 10% at ^10^B concentrations of the order of 100 ppm. Clinical boron concentrations of this order have been previously reported in the literature^31,32^. Below this concentration, it would be necessary to increase detection efficiency by utilising multiple detector heads around the target.

Together with the results published in our previous paper^18^ (which explored neutron capture discrimination with boron present in the target but not in the detector), we have now comprehensively evaluated the expected gamma-ray spectra in simulation for all relevant conditions (no boron in target or detector, boron in target but not detector, and boron in both target and detector), and experimentally evaluated the case where boron is present in both the target and detector PCB.

## Conclusions

In this study, a prototype scintillator-based detector was used to measure changes in the photon spectrum due to the addition of a boron insert in a PMMA target subject to irradiation by helium and carbon ion beams. Increases in the area of the 478 keV ^10^B thermal neutron capture peak of 26 ± 2% and 45 ± 7% were observed for the helium and carbon ion beams, respectively. When the experiment was modelled using the Geant4 Monte Carlo toolkit, corresponding increases in area of the 478 keV peak of 57 ± 1% and 45 ± 1% were obtained for simulated helium and carbon ion irradiation, respectively.

From the simulation, it was estimated that more than 65% of 478 keV photons originated from ^10^B thermal neutron capture occurring in the PCB of the detector electronics, which is more than double the proportion originating from neutron captures in the boron insert. This finding highlights the importance of neutron shielding for the detector, and strongly supports a recommendation to use strictly boron-free materials in the detector electronics, especially the PCB.

Finally, an increase in the number of neutron capture photons arriving at the detector has been measured with increasing concentration of ^10^B. The increase is approximately linear up to a ^10^B concentration of approximately 20,000 ppm, where the signal reaches a plateau due to all of the thermal neutron interactions having been captured. With the addition of temporal windowing, it is expected that a single detector system should be able to detect neutron capture events at ^10^B concentrations as low as 100 ppm, while a multi-detector system would be required for lower concentrations.

The experimental and simulation work performed in this study provides important proof of concept for our proposed thermal neutron capture quantification scheme, and is an essential step in the future development of an improved prototype detector for quantifying boron thermal neutron capture in particle therapy featuring both temporal and spectral windowing. This will ultimately enable the development of a SPECT-like neutron capture imaging system which will provide an essential quality assurance mechanism for NCEPT.

### Supplementary Information

Supplementary Information.

## Data Availability

All data generated or analysed during this study are included in this published article or are available from the corresponding author on reasonable request.
