# Supplementary material for: First experimental demonstration of real-time neutron capture discrimination in helium and carbon ion therapy
Source: Sci Rep. 2024 Jan 31;14:2601. doi: 10.1038/s41598-024-52162-9 (PMC10831067; doi:10.1038/s41598-024-52162-9)
Supplement: Supplementary file 1 — Supplementary Information. [file 41598_2024_52162_MOESM1_ESM.pdf]

First experimental demonstration of real-time neutron capture discrimination in helium and carbon ion therapy: Supplementary Materials

Marissa Kielly[1,2], Anita Caracciolo[3,4], Andrew Chacon[1], James Vohradsky[2], Davide Di Vita[3,4], Akram Hamato[5], Hideaki Tashima[5], Daniel R. Franklin[6], Taiga Yamaya[5], Anatoly Rosenfeld[2], Marco Carminati[3,4], Carlo Fiorini[3,4], Susanna Guatelli[2], and Mitra Safavi-Naeini[1,*]

[1]Australian Nuclear Science and Technology Organisation (ANSTO), Lucas Heights, Australia
[2]Centre for Medical Radiation Physics, University of Wollongong, Wollongong, Australia
[3]Dipartimento di Elettronica, Informazione e Bioingegneria, Politecnico di Milano, Milano, Italy
[4]Istituto Nazionale di Fisica Nucleare (INFN), Sezione di Milano, Milano, Italy
[5]Imaging Physics Group, Department of Advanced Nuclear Medicine Sciences, National Institutes for Quantum Science and Technology (QST), Japan
[6]School of Electrical and Data Engineering, University of Technology Sydney, Sydney, Australia
[*]mitras@ansto.gov.au

S1 Supplementary Methods

S1.1 Monte Carlo physics models

A list of Geant4 physics models used in all Geant4 simulations conducted in this study is presented in Table S1.

For the physics of MCNP v6.2, the Bertini Intranuclear Cascade (INC) physics model was used to describe all hadronic processes. A default lower energy limit cut-off of 1 keV was set for all particles, with the exception of neutrons, for which the lower energy limit was set at 0 keV. Neutron tracking and capture processes were set to be in full analog mode with no variance reduction. Neutron interactions were described using continuous energy datasets, largely sourced from the ENDF/B-VII.1 libraries. The Neutron Capture Ion Algorithm (NCIA) describes the production of light and heavy ions from neutron capture and was only used when the production of these ions were not modelled with available nuclear data tables.

S1.2 Simulation configuration

The detailed configuration used for the comparison of neutron production and transport in MCNP and Geant4 is shown in Figure S1. The neutron spectra at two planes, one for neutrons leaving the phantom at the surface facing towards the detector and one at the surface of the detector, were scored for both Monte Carlo toolkits. These planes are given in the schematic in pink. The phantom and detector configuration is the same as the main simulation in this study, without the presence of a boron insert. The primary ions generated on the surface of the phantom are also the same SOBP60 ^{4}He and ^{12}C beams.

S2 Supplementary Results

The neutron spectra for the Geant4 and MCNP simulations are given in Figure S2 and Figure S3 for the neutron fluence exiting the phantom and reaching the detector face, respectively. Error bars are given as 2 standard errors for Geant4 results only, with an average plotted over 20 populations of 1×10^{8} histories for the helium beam and 5×10^{7} for carbon.

The number of thermal neutrons is calculated based on the total neutron fluence under the peak from 0.002 eV to 0.44 eV for each case and given in Table S2.

Table S1. Physics models used in all simulations.

**Interaction**	**Energy Range**	**Geant4 Model**
Electromagnetic Interactions	N/A	`G4EmStandardPhysics_option4`
Radioactive Decay	N/A	G4RadioactiveDecayPhysics
Particle Decay	N/A	G4Decay
Hadron Elastic	0–100 TeV	G4HadronElasticPhysicsHP
Ion Inelastic	0–110 MeV<br>100 MeV–10 GeV<br>9.99 GeV–1 TeV	Binary Light Ion Cascade<br>BIC<br>FTFP
Neutron Capture	0–20 MeV<br>19.9 MeV–100 TeV	NeutronHPCapture<br>nRadCapture
Neutron Inelastic	0–20 MeV	NeutronHPInelastic
Neutron Elastic	0 eV–20 MeV<br>20 MeV–100 TeV	NeutronHPElastic<br>hElasticCHIPS
Proton Inelastic	0–9.9 GeV	Binary Cascade

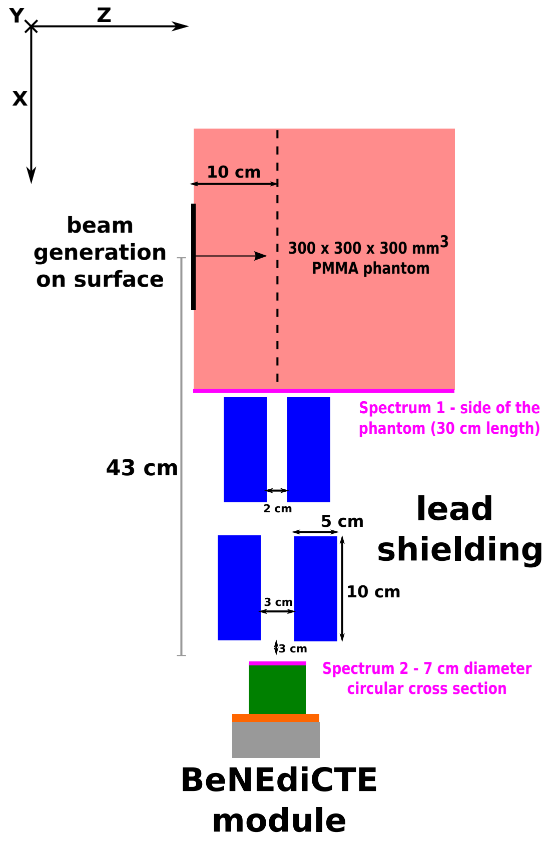

Figure S1. Simulation configuration for the MCNP/Geant4 comparison study.

	Neutrons leaving the phantom	Neutrons at the detector face
^{4}He	0.89	0.72
^{12}C	1.12	0.97

Table S2. Ratio of total thermal neutrons predicted by MCNP to Geant4

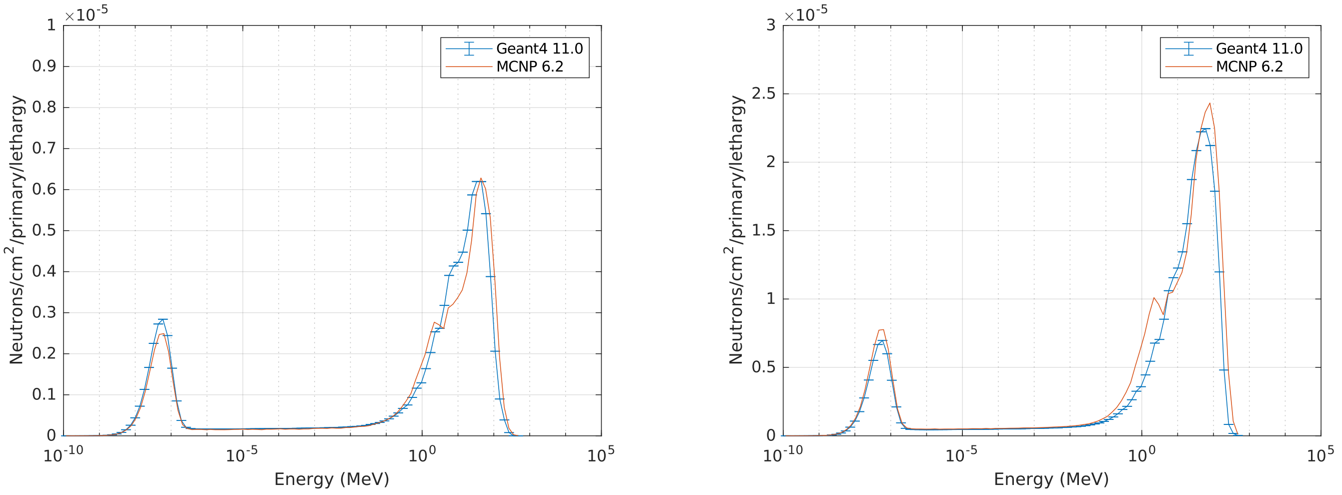

(a) ^{4}He

(b) ^{12}C

Figure S2. Neutron spectra for all neutrons leaving the surface of the phantom.

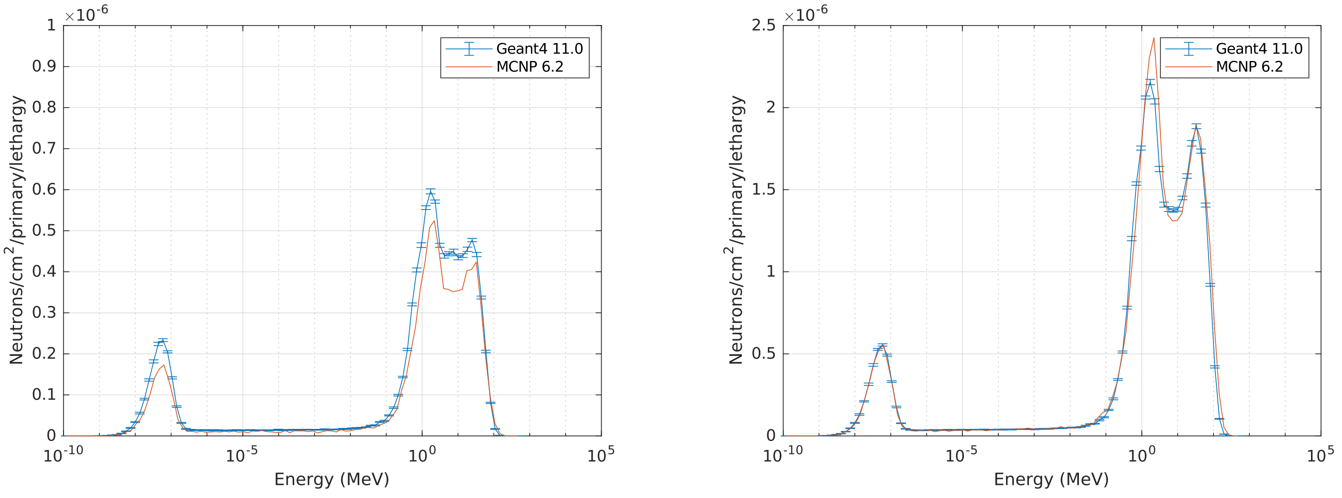

(a) ^{4}He

(b) ^{12}C

Figure S3. Neutron spectra for all neutrons incident on the face of the detector.
